# Nash Equilibria in the Response Strategy of Correlated Games

**DOI:** 10.1038/s41598-018-36562-2

**Published:** 2019-02-20

**Authors:** A. D. Correia, H. T. C. Stoof

**Affiliations:** Institute for Theoretical Physics and Center for Complex Systems Studies, Utrecht University, P.O. Box 80.089, 3508 TB Utrecht, The Netherlands

## Abstract

In nature and society, problems that arise when different interests are difficult to reconcile are modeled in game theory. While most applications assume that the players make decisions based only on the payoff matrix, a more detailed modeling is necessary if we also want to consider the influence of correlations on the decisions of the players. We therefore extend here the existing framework of correlated strategies by giving the players the freedom to respond to the instructions of the correlation device by probabilistically following or not following its suggestions. This creates a new type of games that we call “correlated games”. The associated response strategies that can solve these games turn out to have a rich structure of Nash equilibria that goes beyond the correlated equilibrium and pure or mixed-strategy solutions and also gives better payoffs in certain cases. We here determine these Nash equilibria for all possible correlated Snowdrift games and we find these solutions to be describable by Ising models in thermal equilibrium. We believe that our approach paves the way to a study of correlations in games that uncovers the existence of interesting underlying interaction mechanisms, without compromising the independence of the players.

## Introduction

Game theory^1^ has been used as a powerful tool to model problems from diverse research areas. In particular, evolutionary game theory^2^ aims to explain why some strategies are selected in the presence of many players^3^. When we are dealing with a system of only two players that are solely informed by a payoff matrix the solutions are very well understood, but when several players are involved it becomes, in spite of that, hard to predict the final equilibrium only from the pair-wise interactions^4^. This has found many applications in biology^5^, economics^6–8^, politics^9^, and social sciences^10,11^. In this respect, a longstanding challenge is to understand when individuals in groups end up cooperating, while their individual, rational strategy would be to defect^12^, such as occurs in the Prisoner’s Dilemma game^13^. This situation is relevant in evolutionary game theory because a number of biological systems can be modeled by this famous game^14^ and as a consequence this issue has received much attention^5,15,16^. Also relevant is the increased understanding of complex networks^17^, which has proved essential to study the multiplayer situation because it allows us to obtain more insight into the effect of the interaction structure on the cooperation of the players on the network^18,19^. Additionally, methods from statistical physics have proved fruitful at studying the phase transition aspects of group behavior. Examples of such methods are Monte Carlo simulations and mean-field techniques, that for instance use Fermi-Dirac statistics to obtain the decisions of the players based on the decisions of their neighbors in the previous round^11,20^. As a natural development, these physics-based techniques have also been applied to players on different kinds of networks^13,18^. For games where the best individual strategy is to defect, this research has shown that cooperation, among other factors, strongly depends on specific game-theoretical parameter values and on network structure^4,20^. If we consider that the players play a Snowdrift game instead, the best individual payoff can sometimes be to cooperate. This explains the occurrence of cooperation as part of a mixed strategy solution^2^, but explaining how it becomes an emergent behavior for a large number of players is still complicated^21^. This game is of particular interest because it has been shown to model biological conflict^22,23^. Interestingly, the optimal payoff for this game is only achieved with communication between the players, and so it is worth exploring if, in certain situations, third-party information might weigh on their decisions.

Games that incorporate extra information in this way are known in the literature as “expanded games” that, in addition to the payoff matrix, include a so-called correlation device, which is an external party that draws with a fixed probability one of the final configurations of the game and informs each of the players separately of what they should play to achieve that configuration^1^. The correlations can either be agreed upon beforehand by the players as a correlated strategy to solve the game or they can be externally imposed. If both players do not have an incentive to not follow the recommendations of the correlation device while their opponent is following the instructions, the players are said to be in correlated equilibrium. Keeping both possible interpretations of the game in mind we are now interested in exploring the following questions. First, is it possible for each player to obtain a better payoff than in correlated equilibrium by having a different strategy over the correlations, other than always complying with the instructions from the correlation device, and that is in a Nash equilibrium? Secondly, in case the initially agreed upon or externally imposed correlations do not allow for a correlated equilibrium, is it still possible for the players to use the correlation device to find a better solution other than returning to the case without correlations, by ignoring the correlation device and using the pure or mixed strategies solutions of the game? To answer these questions, we introduce a new kind of strategy, which consists of a probability distribution over following or not following the correlation device.

With the ultimate goal to further advance the application of games to real-life situations and to explain group behavior from the pair-wise interactions, we focus in this paper on two-player games that are described by a payoff matrix and a correlation device, both of which are specified beforehand and remain unchanged during the game. In particular, the correlations can also be out of correlated equilibrium. These games we call “correlated games” from now on and they can be solved by finding the Nash equilibria in the response probabilities to follow the instructions of the correlation device. By giving an added degree of freedom to the players that are already subject to correlations, our results represent a considerable improvement on the analysis of these kind of games. For instance, our approach shows that a correlated equilibrium is always locally stable, but does not have to be stable globally, that is, there can exist different Nash equilibria that have a higher payoff. For practical applications, our treatment is promising because we expect that better outcomes can be achieved if we do not assume from the start that the players act solely based on their individual payoffs but consider as well that the decisions can be informed by underlying correlations in the system. While the treatment is generalizable to any game, we study its effects on the Snowdrift game for two players. Because we are now dealing with direct interactions between the players, we can readily express our results also in terms of probabilities given by a Boltzmann distribution.

## Correlated Games

**Figure 1:**
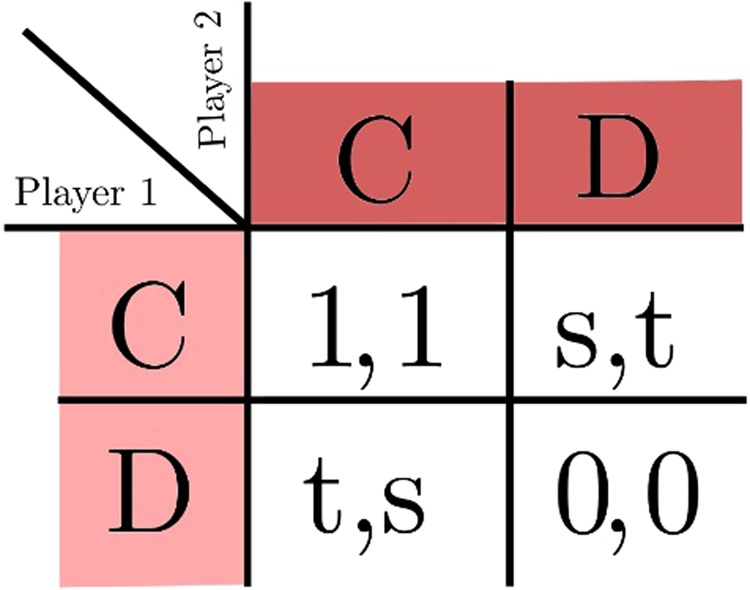
Normalized payoff table for two-by-two, symmetric games.

We consider symmetric, two by two games. The players have access to the payoff matrix in Fig. 1, that settles how much reward each player receives given the actions of all the players. Player 1 receives the payoffs on the left side of the comma, while player two receives the payoff on the right side of the comma, dependent on the two strategies chosen. The different games are defined by the range of the parameters: the Harmony game has 0 < *s* < 1 and 0 < *t* < 1; the Stag-Hunt game has −1 < *s* < 0 and 0 < *t* < 1; the Prisoner’s Dilemma has −1 < *s* < 0 and 1 < *t* < 2; and the Snowdrift game, also known as Chicken or Hawk-Dove game, has 0 < *s* < 1 and 1 < *t* < 2. Depending on the game being played, the players decide on the best strategy based on how much they will win given all the possible strategies of the adversaries. For the uncorrelated case, the objective is to maximize the expected payoff by assigning a probability *P*_*C*_ to playing *C* (to cooperate), so that *D* (to defect) is played with the probability 1−*P*_*C*_. A Nash equilibrium^24^ is reached for a strategy, i.e., a value of *P*_*C*_, that none of the players wants to deviate from. If *P*_*C*_ is 0 or 1, it is a pure Nash equilibrium, otherwise it is a mixed strategy equilibrium. The mixed strategy solution is of particular importance in the Snowdrift games. This game has two pure Nash equilibria in which the players adopt opposite pure strategies, but these cannot be reached without introducing correlations between the players. Therefore, the best solution is a mixed strategy Nash equilibrium, where the probability of playing *C* for each player is ${P}_{C}^{\ast }=s/(t+s-1)$.

This solution for the Snowdrift game is the best that the players can do without communicating, but, for most parameters, better results are obtained if they can both play opposite strategies systematically. This, however, requires the introduction of correlations between the players. To illustrate this, we consider the extreme example of a simple traffic light on a cross-road. The cross-road can also be described as a Snowdrift game, where the best payoffs are obtained when one of the drivers decides to stop (to cooperate) and the other decides to go (to defect). Since the players cannot communicate, a traffic light is needed to achieve the optimal situation. The traffic light can be seen as the correlation device, which assigns a publicly known probability *p*_*μν*_ to a certain state *μν*, with Greek indices taking the values *C* and *D*. In this particular example, the device assigns equal probabilities to the states *CD* and *DC*, while assigning zero probability to *CC* and *DD*. Since the correlation is very strong and there is a big penalty if both players defect simultaneously, the players always want to follow the correlation device, and the game is thus in a “correlated equilibrium”. A general correlation device, however, assigns non-zero probabilities to all the possible outcomes of a game. If this is the case for the states with low payoff, the question arises whether always following is the best strategy for the players. According to the existing theory^25^, they should always follow the correlation device if they are in correlated equilibrium, and they should fall back to the uncorrelated mixed-strategy solution otherwise. This ensures that the probabilities in correlated equilibrium coincide with the final distribution of outcomes, such that they represent the actual statistics of the game. Thus, if the players always follow the instructions from the correlation device, this distribution perfectly describes the actions of the players. To show that this is not the complete picture, we now allow the players to deviate from the instructions of the correlation device in a controlled manner. This forms a new game, a “correlated game”, where the players now play against the correlation device, having an option to follow or not follow the received instructions.

## Response Strategy

To solve the correlated game, a “response strategy” involving the decisions to follow or not to follow the instructions arises. The probablities to act in either way become the new actions that the players can take, while they are still not able to comunicate. To implement this, each player i can follow with probability ${P}_{F\mu }^{i}$, and thus not follow with probability ${P}_{NF\mu }^{i}=1-{P}_{F\mu }^{i}$ the instruction *μ* that they receive. These probabilities we call the “response probabilities”. The renormalized probability ${p}_{\mu \nu }^{R}$ that a certain final state *μν* is reached is given by the sum over the initial probability distribution weighted by the probability that the initial states *μ*′*ν*′ gets converted to a specific final state *μν* through the players’ response. Hence^26–29^

1 $${p}_{\mu \nu }^{R}=\sum _{\mu ^{\prime} ,\nu ^{\prime} }\,{P}_{\mu \leftarrow \mu ^{\prime} }^{1}{P}_{\nu \leftarrow \nu ^{\prime} }^{2}{p}_{\mu ^{\prime} \nu ^{\prime} },$$

with ${P}_{\mu \leftarrow \mu ^{\prime} }^{i}$ the probability that player i is told to play *μ*′ but plays *μ*. As an example, the probability that the final state is *CC* is now

2 $${p}_{CC}^{R}={P}_{FC}^{1}{P}_{FC}^{2}\,{p}_{CC}+{P}_{FC}^{1}{P}_{NFD}^{2}{p}_{CD}+{P}_{NFD}^{1}{P}_{FC}^{2}\,{p}_{DC}+{P}_{NFD}^{1}{P}_{NFD}^{2}{p}_{DD}\mathrm{.}$$

The expected payoff of a player is given by the payoffs averaged over the renormalized probabilities, which depends linearly on the response probabilities of that player as

3 $$\langle {u}^{i}\rangle =\sum _{\mu ,\nu }\,{u}_{\mu \nu }^{i}{p}_{\mu \nu }^{R}={C}_{C}{P}_{FC}^{i}+{C}_{D}{P}_{FD}^{i}+{C}_{E},$$

with ${u}_{\mu \nu }^{i}$ the payoff of player i in the state *μν*. The coefficients *C*_*C*_, *C*_*D*_ and *C*_*E*_ arise when we group the terms in the sum according to their dependence on the probabilities of player *i*. They depend linearly on the initial correlation probabilities and on the response probabilities of the opponent. In essence, *C*_*C*_, *C*_*D*_ tell us how the average payoff of one player changes when this player changes their response probabilities.

A Nash equilibrium in the response strategy is achieved if there is no incentive for player i to change the probabilities ${P}_{F\mu }^{i}$. This is achieved by imposing that the slope of the expected payoff with respect to each response probability, either *C*_*C*_ or *C*_*D*_ in the evaluation of the total player’s payoff, is zero, unless the equilibrium response probability is 0 or 1, in which case the slope should be negative or positive, respectively. The intuition is that equilibrium is reached when the payoff of the players cannot be improved anymore by changing their own response probabilities while keeping those of the other players fixed at the equilibrium values. The independence of this analysis separately for each response probability represents Bayes rationality^25^ of the players towards the final states given the initial information that they receive.

As a result, there are three possible Nash equilibria for each response probability: *P*_*Fμ*_ = 0, *P*_*Fμ*_ = 1 and 0 < *P*_*Fμ*_ < 1. In our two by two games, this amounts to nine possible types of equilibrium response strategies, but which ones are actually realized depends on the payoff parameters. We find that the conditions where “always follow”, i.e., *P*_*Fμ*_ = 1 is a stable solution, correspond to the Bayes rational conditions of the correlated equilibrium, indicating that this is only one possible response equilibrium. However, each renormalized set of probabilities generates a new correlated game for which the response equilibrium exactly matches a correlated equilibrium, from which the players by definition indeed do not want to deviate. Using the slopes *C*_*C*_ and *C*_*D*_ to evaluate the Nash equilibria, each of the response probabilities has to be subject to one of the three above-mentioned conditions simultaneously. To guarantee that none of the players wants to deviate, each slope is calculated assuming that the response probabilities of the other players are in equilibrium, such that a self-consistent solution is obtained (see Supplementary Material).

## Ising Model

Another way of representing any symmetric, and thus possibly correlated, probability distribution over the four possible states is through a Boltzman distribution of an Ising system. Let each player be represented by a particle with spin, and let *C* and *D* correspond to the spin states “up” and “down”, such that *μ*, *ν* ∈ {↑, ↓}. We can rewrite the probabilities as

4 $${p}_{\mu \nu }=\frac{{e}^{-\beta {H}_{\mu \nu }}}{Z},$$

with β the inverse of a generalized thermal energy, *H*_*μν*_ the Hamiltonian of the Ising system, decomposable into the sum of a constant term that can be absorbed into the partition function, an Ising term and a Zeeman term^30^, and $Z={\sum }_{\mu ,\nu }{e}^{-\beta {H}_{\mu \nu }}$ the partition function.

Having found the optimal response probabilities, we can now incorporate them in the Ising model that effectively describes the final statistics of the game. Using the language of statistical physics, the response probabilities in equilibrium can be written as

5 $${P}_{\mu \leftarrow \mu ^{\prime} }^{i}=\frac{{e}^{-\beta {B}_{\mu \leftarrow \mu ^{\prime} }^{i}}}{{Z}_{\mu ^{\prime} }^{i}},$$

with the partition function given by

6 $${Z}_{\mu ^{\prime} }^{i}=\sum _{\mu }\,{e}^{-\beta {B}_{\mu \leftarrow \mu ^{\prime} }^{i}},$$

where ${B}_{\mu \leftarrow \mu ^{\prime} }^{i}$ are the appropriate energies that are explicitly given in the Methods section. The renormalized correlated probabilities, by eq. 4, are written in closed form as

7 $${p}_{\mu \nu }^{R}=\frac{{e}^{-\beta {H}_{\mu \nu }^{R}}}{{Z}^{R}}\mathrm{.}$$

Using eqs 4, 5 and 7, we are able to describe the renormalized Ising Hamiltonian as

8 $${H}_{\mu \nu }^{R}=-\,\frac{1}{\beta }\,{\rm{l}}{\rm{n}}(\sum _{\mu {\rm{^{\prime} }}\nu {\rm{^{\prime} }}}\,{Z}_{-\mu {\rm{^{\prime} }}}^{1}{Z}_{-\nu {\rm{^{\prime} }}}^{2}\,{e}^{-\beta ({B}_{\mu \leftarrow \mu {\rm{^{\prime} }}}^{1}+{B}_{\nu \leftarrow \nu {\rm{^{\prime} }}}^{2}+{H}_{\mu {\rm{^{\prime} }}\nu {\rm{^{\prime} }}})}).$$

The minus sign indicates the opposite play, i.e., −*C* = *D* and −*D* = *C*, or the corresponding interchange between spin states “up” and “down”. The renormalized Hamiltonian is obtained by the players choosing the energy parameters ${B}_{\mu \leftarrow \mu ^{\prime} }^{i}$ independently, which in this formalism is what allows the players to have complete control over the final probabilities. How eqs 5 and 8 are obtained is explicitly explained in the Methods section. Note that if eq. 8 is interpreted as a renormalization-group transformation, the Nash equilibria correspond to the fixed points of this transformation^30^.

## Results

**Figure 2:**
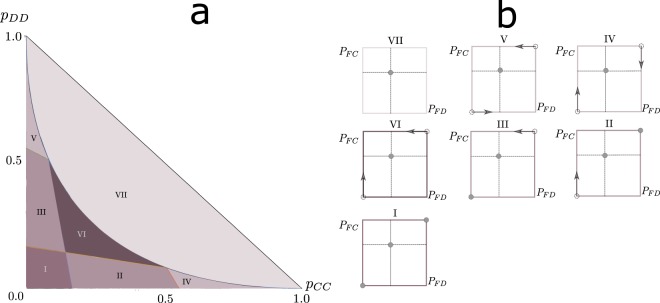
Symmetric correlations and associated equilibrium response strategies for the Snowdrift game with *s* = 0.5 and *t* = 1.2. (**a**) Illustration of all correlated Nash equilibria of the Snowdrift game in the *P*_*CC*_ − *P*_*DD*_ plane, for parameters representative of *s* > *t* − 1. (**b**) Schematic representation of the equilibrium value of the response probabilities in the *P*_*FD*_ − *P*_*FC*_ plane that can be found in each region enumerated in (**a**).

We studied the response strategies of the Snowdrift game for all initial (symmetric) correlation probabilities and show the results in Fig. 2. The lines that separate each region in 2a are obtained by imposing a particular sign on a slope of a response probability and using the associated value for that probability. Between each straight line and the curved line, given by ${p}_{DD}=1+{p}_{CC}-2\sqrt{{p}_{CC}}$, there exists a solution with one of the slopes strictly greater or smaller than zero and the other one equal to zero, such that one of the response probabilities is 0 or 1 and we can find a value for the other probability that lies between these values. Each response probability associated with a zero slope can have values that range between the value of the other probability in the extreme and its associated mixed-strategy solution (due to the limiting condition that *p*_*DD*_ ≤ 1−*p*_*CC*_), represented in 2b by the dotted line. Bellow the lines, both slopes have the same sign and both probabilities are in the same extreme of the interval. The arrows in 2b depict how the value of the probabilities change as we move from the straight lines towards the curved line, which when reached sends the probabilities to the uncorrelated mixed-strategy solution that is always a solution in all regions. The upper line that delimitates regions I and II and the rightmost line that delimitates region II are the lines that arise when both slopes are positive. They correspond to the correlated equilibrium conditions. The intersection of these two lines is the mixed-strategy solution when written as a response strategy. Thus in these two regions the correlated equilibrium is a solution, although it is not unique or always optimal in payoff. Moreover, there are regions where the correlated equilibrium is unstable but other solutions exist that make use of the correlations to increase the payoff. For *s* < *t*−1 the results are similar, but there occurs a swapping among the lines: the rightmost boundary of region I becomes the leftmost, and the top boundary of region III becomes the bottom one, which changes the equilibria in regions II, III and VI correspondingly. When *s* = *t*−1 these boundaries overlap and these regions disappear. In addition to the expected existence of a correlated equilibrium region, where interestingly also other Nash equilibria exist, we see other regions with other types of equilibria that use correlations to optimize the payoff. The mixed strategy of the uncorrelated Snowdrift game is always an equilibrium solution in every region.

**Figure 3:**
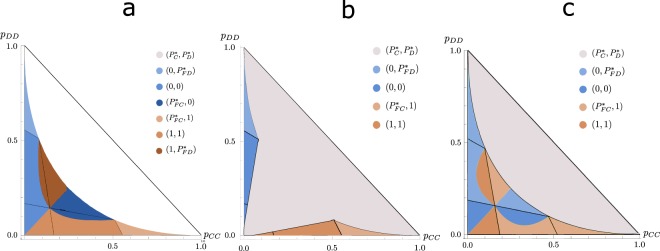
Equilibrium response strategies with highest payoff per region, for different parameters. (**a**) Equilibria corresponding to highest payoff by region, for *s* = 0.5 and *t* = 1.2 (*s* > *t* − 1), without the mixed-strategy solution. (**b**) Equilibria corresponding to highest payoff, for the same parameters as (**a**), including the mixed-strategy solution. (**c**) Equilibria corresponding to highest payoff by region, for *s* = 0.23 and *t* = 1.5 (*s* < *t* − 1), including the mixed-strategy solution. The existing equilibrium solutions are compared within a region, according to the description in Fig. 2. The darkening of the colors represents a higher absolute value of the payoff when compared to the best payoffs of the neighboring regions, but the actual value changes within the region, except for the mixed-strategy solution, of which the is constant. All the payoffs corresponding to the best solution are continuous to one another.

To choose the best response for each region, we compare in Fig. 3 the payoffs of all the possible response equilibria. As the parameters vary in the figures, the blue solution in region II and the orange solution in region III either increases or decreases in area. In Fig. 3b we see that the mixed strategy can be the best solution for a game with strong off-diagonal correlations, but this is not generally the case for all parameters. In the regions where the correlated equilibrium exists, there is, for certain initial probabilities, a better alternative solution. All the renormalized games will correspond to final probabilities where the correlated equilibrium is the best solution, such that the players do not want to deviate anymore from their chosen strategy. Because the mixed-strategy payoff is a constant, we see that correlations always provide a payoff that is at least as good.

## Discussion

The introduction of the possibility to follow or not follow the instructions from a correlation device, which we called a response strategy, in correlated games opens up several new features. We showed that the correlated equilibrium is only a particular response equilibrium, but that other Nash equilibria exist. These new equilibria renormalize to a correlated equilibrium even if the initial game is out of correlated equilibrium, showing that the players even then can still use the correlations to achieve a better payoff. The extra information in the correlations is two-fold: either the final distributions of outcomes informs us about an underlying correlation structure, or the players can independently improve on externally imposed initial correlations, motivated by stability and payoff maximization. Regarding evolutionary game theory, the correlated evolutionary stable strategy^27,31^ is, similarly, only one equilibrium where the agents always follow the correlations, which suggests that the evolutionary stable solution is not unique. The fact that the correlations might not completely determine the actions of the players but merely inform them is an essential feature in our model that allows the encoding of the choice of the players.

While extensive research has been done to study how humans behave when they play games on networks, particularly through simulations^10,32,33^ and experiments^32,34,35^, we hope to provide analytical insight into these systems, since for the two-player game the renormalized probabilities of the optimal Nash equilibrium are equivalent to a two-site Ising model in a magnetic field. To this effect, we show how the players can introduce an energy parameter to change the Hamiltonian to effectively obtain the renormalized probabilities. This provides a new way in which statistical physics methodology can be used to model a simple network with interactions, adding to the existing research that applies physics to phenomena that involve several agents^4,11,36,37^, with particular emphasis to the applications in game theory^13,37,38^. Bridging the gap between games with and without correlations will also prove useful to better model decision-making, since the response probabilities allow us to include interactions between agents that influence their decisions. It is also worth mentioning other efforts to include the possibility of additional information and voluntary participation, such as coevolutionary games^39^, in particular where the players have a third strategy that represents the option to abstain from playing^40^, spatial games where the players are assigned a probability to abstain from playing^41^ and multilayer networks^42^. It remains an interesting open question how well our particular model would describe the non-local network effects and this constitutes future research.

## Methods

### Slope analysis for the Snowdrift Game

In eq. 3 we have an expression for the expected payoff of player 1, with the coefficients *C*_*C*_ and *C*_*D*_. To make the various dependencies clearer, we now denote these explicitly as ${C}_{C}({p}_{CC},\,{p}_{DD},\,{P}_{FC}^{2},\,{P}_{FD}^{2})$ and ${C}_{D}({p}_{CC},\,{p}_{DD},\,{P}_{FC}^{2},\,{P}_{FD}^{2})$, respectively.

For the Snowdrift game, which is a symmetric game, it is natural to assume also a symmetric probability distribution, so *p*_*CD*_ = *p*_*DC*_ = (1 − *p*_*CC*_ − *p*_*DD*_)/2. When both slopes are positive, the equilibrium will be at ${P}_{FC}^{1}=1$ and ${P}_{FD}^{1}=1$, and due to the symmetry of the game, player 1 will not want to change these probabilities when player 2 has the same equilibrium probabilities. To reach equilibrium, the conditions thus become

9 $$\{\begin{array}{ccc}{C}_{C}({p}_{CC},\,{p}_{DD},\,1,\,1) &  >  & 0,\\ {C}_{D}({p}_{CC},\,{p}_{DD},\,1,\,1) &  >  & 0\end{array}$$

These conditions are equivalent to the correlated equilibrium conditions. Similarly, a second kind of equilibrium is reached when the slopes are negative and the response probabilities are zero, i.e.,

10 $$\{\begin{array}{ccc}{C}_{C}({p}_{CC},\,{p}_{DD},\,0,\,0) &  <  & 0,\\ {C}_{D}({p}_{CC},\,{p}_{DD},\,0,\,0) &  <  & 0\end{array}$$

A third type of equilibrium exists when both slopes equate to zero

11 $$\{\begin{array}{ccc}{C}_{C}({p}_{CC},\,{p}_{DD},\,{P}_{FC}^{1\ast },\,{P}_{FD}^{1\ast }) & = & 0,\\ {C}_{D}({p}_{CC},\,{p}_{DD},\,{P}_{FC}^{1\ast },\,{P}_{FD}^{1\ast }) & = & 0,\end{array}$$

for which the solution coincides with the mixed-strategy equilibrium solution, with ${P}_{FC}^{1\ast }={P}_{FC}^{2\ast }={P}_{C}^{\ast }$ and ${P}_{FD}^{1\ast }={P}_{FD}^{2\ast }={P}_{D}^{\ast }$. The last type of equilibria comes in four possible guises, consisting of one of the conditions being zero, while the other is strictly positive or negative. For instance, we can have

12 $$\{\begin{array}{ccc}{C}_{C}({p}_{CC},\,{p}_{DD},\,1,\,{P}_{FD}^{1\ast }) &  >  & 0,\\ {C}_{D}({p}_{CC},\,{p}_{DD},\,1,\,{P}_{FD}^{1\ast }) & = & 0,\end{array}$$

If we calculate the specific value of the response probability using the first line of eq. 12 and substitute this value in the second line, a new condition arises for symmetric games, namely

13 $${p}_{DD} < 1+{p}_{CC}-2\sqrt{{p}_{CC}}\mathrm{.}$$

Under this condition, and *p*_*DD*_ ≤ 1−*p*_*CC*_, each equilibrium response probability that has a zero slope in the expected payoff, has a limited range of possible values, which, depending on the sign of the associated inequality, goes from 1 or 0 to ${P}_{C}^{\ast }$ or ${P}_{D}^{\ast }$. Each of the four possible combinations correspond to one of the four response equilibria of this kind. The solution of the example given above is ${P}_{FC}^{1\ast }=1$ and ${P}_{D}^{\ast }$ $< {P}_{FD}^{1\ast } < 1$, with the specific value of ${P}_{FD}^{1\ast }$ depending on the initial correlated probabilities.

Due to the value of the Snowdrift game’s payoff parameters, the two other conditions that would arise from having the two conditions with opposite strict inequalities do not have solutions, so only seven equilibria exist in total for this game.

Note that in a game-theoretical notation, each of the above equilibrium conditions can be summarized as

$$\sum _{\mu ,\nu ,\nu ^{\prime} }\,{u}_{\mu \nu }^{1}{P}_{\mu \leftarrow \mu ^{\prime} }^{1\ast }{P}_{\nu \leftarrow \nu ^{\prime} }^{2\ast }{p}_{\mu ^{\prime} \nu ^{\prime} }\ge \sum _{\mu ,\nu ,\nu ^{\prime} }\,{u}_{\mu \nu }^{1}{P}_{\mu \leftarrow \mu ^{\prime} }^{1}{P}_{\nu \leftarrow \nu ^{\prime} }^{2\ast }{p}_{\mu ^{\prime} \nu ^{\prime} }\mathrm{.}$$

These comprehend two conditions, one for every value of *μ′*. Summing over these shows that the expected payoff of player 1 cannot be improved by deviating from the equilibrium strategy, which is the requirement for a Nash equilibrium. The stronger statement that both conditions are satisfied separately expresses the Bayes rationality of player 1.

### Renormalized Ising Model

To insert the response probabilities in the Ising model, each reaction from the players will have a Zeeman-like energy: either $-{B}_{\mu }^{i}$ if they follow *μ*, or $+\,{B}_{\mu }^{i}$ if they do not follow:

14 $${B}_{\mu \leftarrow \mu {\rm{^{\prime} }}}^{i}=-\,{\delta }_{\mu \mu {\rm{^{\prime} }}}{B}_{\mu }^{i}+(1-{\delta }_{\mu \mu {\rm{^{\prime} }}}){B}_{\mu }^{i}.$$

We can then rewrite the response probabilities as

15 $${P}_{\mu \leftarrow \mu ^{\prime} }^{i}=\frac{{\delta }_{\mu \mu ^{\prime} }{e}^{\beta {B}_{\mu ^{\prime} }^{i}}+\mathrm{(1}-{\delta }_{\mu \mu ^{\prime} }){e}^{-\beta {B}_{\mu ^{\prime} }^{i}}}{{Z}_{\mu ^{\prime} }^{i}}=\frac{{e}^{-\beta {B}_{\mu \leftarrow \mu ^{\prime} }^{i}}}{{Z}_{\mu ^{\prime} }^{i}},$$

with ${Z}_{\mu ^{\prime} }^{i}$ as given in eq. 6. Using eq. 1, we calculate the renormalized Ising energies:

$${H}_{\mu \nu }^{R}=-\,\frac{1}{\beta }\,{\rm{l}}{\rm{n}}({Z}^{R}\,{p}_{\mu \nu }^{R})=-\,\frac{1}{\beta }\,{\rm{l}}{\rm{n}}(\sum _{\mu {\rm{^{\prime} }},\nu {\rm{^{\prime} }}}\,{P}_{\mu \leftarrow \mu {\rm{^{\prime} }}}^{1}{P}_{\nu \leftarrow \nu {\rm{^{\prime} }}}^{2}{p}_{\mu {\rm{^{\prime} }}\nu {\rm{^{\prime} }}})-\frac{1}{\beta }\,{\rm{l}}{\rm{n}}({Z}^{R})$$

Introducing eq. 16 and the initial correlation probabilities with energies *H*_*μ′ν′*_, with associated partition function *Z*, we get a simplified version of the renormalized energies:

16 $$\begin{array}{ccc}{H}_{\mu \nu }^{R} & = & -\frac{1}{\beta }\,{\rm{l}}{\rm{n}}(\frac{{Z}^{R}}{Z{Z}_{C}^{1}{Z}_{D}^{1}{Z}_{C}^{2}{Z}_{D}^{2}})-\frac{1}{\beta }\,{\rm{l}}{\rm{n}}({Z}_{D}^{1}{Z}_{D}^{2}{e}^{-\beta ({B}_{\mu \leftarrow C}^{1}+{B}_{\nu \leftarrow C}^{2}+{H}_{CC})}\\  &  & +\,{Z}_{D}^{1}{Z}_{C}^{2}{e}^{-\beta ({B}_{\mu \leftarrow C}^{1}+{B}_{\nu \leftarrow D}^{2}+{H}_{CD})}+\,{Z}_{C}^{1}{Z}_{D}^{2}{e}^{-\beta ({B}_{\mu \leftarrow D}^{1}+{B}_{\nu \leftarrow C}^{2}+{H}_{DC})}\\  &  & +\,{Z}_{C}^{1}{Z}_{C}^{2}{e}^{-\beta ({B}_{\mu \leftarrow D}^{1}+{B}_{\nu \leftarrow D}^{2}+{H}_{DD})}).\end{array}$$

The first term in eq. 17 drops out because we have that

17 $${Z}^{R}=Z{Z}_{C}^{1}{Z}_{D}^{1}{Z}_{C}^{2}{Z}_{D}^{2}\mathrm{.}$$

Hence, we can rewrite ${H}_{\mu \nu }^{R}$ as in eq. 8. The fact that the renormalized partition function is a product of the various partition functions expresses that the actions of the players enter the renormalized Hamiltonian in an independent manner.

## Electronic supplementary material

Supplementary Material of Nash Equilibria in the Response Strategy of Correlated Games
