# Supplementary material for: Nash Equilibria in the Response Strategy of Correlated Games
Source: Sci Rep. 2019 Feb 20;9:2352. doi: 10.1038/s41598-018-36562-2 (PMC6382789; doi:10.1038/s41598-018-36562-2)
Supplement: Supplementary file 1 — Supplementary Material of Nash Equilibria in the Response Strategy of Correlated Games [file 41598_2018_36562_MOESM1_ESM.pdf]

Suplementary Material
Nash Equilibria in the Response Strategy of Correlated Games

A.D. Correia* and H.T.C. Stoof
Institute for Theoretical Physics and Center for Complex Systems Studies, Utrecht University, P.O. Box 80.089, 3508 TB, Utrecht, The Netherlands.
(Dated: November 20, 2018)

BACKGROUND ON GAME THEORY

Strategic-Form Games

A strategic form game is defined by three elements: the finite set $\mathcal{I}$ of I players, with $\mathcal{I} = \{1, 2, ..., I\}$; the pure-strategy space S_i for each player $i \in \mathcal{I}$, representing the plays that each player has available; and the payoff functions $u_i(s_i, s_{-i})$, denoting the gain of player i if he plays $s_i \in S_i$ and the other players, denoted by $-i$, play $s_{-i} \in S_{-i}$. Besides the pure strategies, the players can play a mixed strategy, in which player i plays the pure strategy s_i with probability $\sigma_i(s_i)$. A pure strategy is a particular case of a mixed strategy, that assigns probability 1 to a certain element of the pure-strategy space.

The players do not have access to what their opponents will play, so the rational player has to consider all his possible moves. Taking this into account, the Nash equilibrium guarantees that each player chooses a strategy from which they do not want to deviate. A mixed strategy profile σ_i^* is the Nash equilibrium if for all players i we have that their average payoff obeys

$$\langle u_i \rangle(\sigma_i^*, \sigma_{-i}^*) \geq \langle u_i \rangle(\sigma_i, \sigma_{-i}^*), \tag{1}$$

with $\sigma_i \in \Sigma_i$ any element of the set of all possible mixed strategy profiles. Since a set of probabilities is convex and compact, it is enough to guarantee that

$$\langle u_i \rangle(\sigma_i^*, \sigma_{-i}^*) \geq \langle u_i \rangle(s_i, \sigma_{-i}^*). \tag{2}$$

for all $s_i \in S_i$.

If the inequality is strict, a pure-strategy Nash equilibrium ensues. In symmetric, two by two, two strategy games, i.e., $S_i = \{C, D\}$ where C denotes to cooperate and D to defect, Nash equilibria are easy to categorize. For the Harmony Game (HG) both players cooperate; in the Prisoner's Dilemma (PD) both defect, and the Stag-Hunt game (SH) has both these two equilibria. The Snowdrift Game (SG), also called Chicken or Hawk-Dove, has two pure strategy Nash equilibria, where one of the players cooperates and the other defects, but these are impossible to achieve, due to the symmetry of the game. The best strategy for this game is a mixed-strategy equilibrium, which assigns an equal probability to cooperate to each player.

In the games that we will analyze, there are only two actions and two players, so $\sigma_i = P_{C_i}$ with $i = 1, 2$, and we rewrite the equilibrium in eq. (1) as

$$\langle u_1 \rangle(P_{C_1}^*, P_{C_2}^*) \geq \langle u_1 \rangle(P_{C_1}, P_{C_2}^*). \tag{3}$$

Expanding, we get in first instance

$$\begin{aligned} &P_{C_1}^* P_{C_2}^* u_1(C,C) + P_{C_1}^*(1 - P_{C_2}^*) u_1(C,D) + (1 - P_{C_1}^*) P_{C_2}^* u_1(D,C) + (1 - P_{C_1}^*)(1 - P_{C_2}^*) u_1(D,D) \\ \geq &P_{C_1} P_{C_2}^* u_1(C,C) + P_{C_1}(1 - P_{C_2}^*) u_1(C,D) + (1 - P_{C_1}) P_{C_2}^* u_1(D,C) + (1 - P_{C_1})(1 - P_{C_2}^*) u_1(D,D). \end{aligned}$$

Subtracting the left-hand side from the right-hand side gives

* Corresponding author: a.duartecorreia@uu.nl

$$\left(P^*_{C_1} - P_{C_1}\right)\left(P^*_{C_2} u_1(C,C) + (1-P^*_{C_2}) u_1(C,D)\right) + \left(P_{C_1} - P^*_{C_1}\right)\left(P^*_{C_2} u_1(D,C) + (1-P^*_{C_2}) u_1(D,D)\right) \geq 0, \tag{4}$$

which after rearrangement of the terms gives the desired result

$$\left(P^*_{C_1} - P_{C_1}\right)\left[P^*_{C_2}\left(u_1(C,C) - u_1(D,C)\right) + (1-P^*_{C_2})\left(u_1(C,D) - u_1(D,D)\right)\right] \geq 0. \tag{5}$$

For this condition to hold, the coefficient of $\left(P^*_{C_1} - P_{C_1}\right)$ has to have the same sign as $\left(P^*_{C_1} - P_{C_1}\right)$ itself. Hence, if P_{C_1} is bigger than $P^*_{C_1}$, the coefficient has to be non-negative, and otherwise, non-positive. The condition in eq. (5) has to hold for all values of P_{C_1} and so, for the case when $P^*_{C_1}$ is not in one of the extremes of the interval, $\left(P^*_{C_1} - P_{C_1}\right)$ can be positive, negative or zero. The only way to ensure that the condition is always true is if it is always zero, meaning that the coefficient has to be zero or

$$P^*_{C_2} u_1(C,C) + (1-P^*_{C_2}) u_1(C,D) = P^*_{C_2} u_1(D,C) + (1-P^*_{C_2}) u_1(D,D). \tag{6}$$

We can thus calculate that the mixed strategy equilibrium probability P^*_{C2} by solving

$$\langle u_1 \rangle (1, P^*_{C_2}) = \langle u_1 \rangle (0, P^*_{C_2}). \tag{7}$$

If $P^*_{C_1}$ is in one of the extremes, then the condition becomes a strict inequality and we obtain a pure strategy equilibrium.

The conditions of the probabilities are those of Kakutani's Theorem, used by Nash to prove the existence of the fixed points that we now know as "Nash Equilibria" [1]. Analyzing the slope of the probability P_{C_1} in the same way as done in our paper for the response probabilities proves the same result.

Correlated Equilibrium

Suppose that the players made some agreement beforehand about what they will play, or that there is some external information that both share even if they do not communicate. This idea is formalized by extending the game with a correlation device. This device draws one of the possible final states, the *true state* $\omega \in \Omega$, with probability $p(\omega)$ and subsequently informs each player of what they should play to achieve the true state ω. Player i then has information $h_i(\omega) \in H_i$, that is, he knows what true states are possible given the information he received. The probability that each of these states is the true state is given by $p(\omega|h_i)$. In the case of the coordination game described above $\Omega = \{CC, CD, DC, DD\}$ and, for example, $h_1(CD) = C$ and $\{\omega|h_1\} = \{CC, CD\}$. This means that if the true state is CD, player 1 is told to play C, at which point he knows that either CC or CD are the possible true states. A correlated strategy $s(\omega)$ represents what pure or mixed strategy each player adopts in the true state ω. The probability distribution $s(\omega)$ is the same as the initial distribution $p(\omega)$ of plays if the players always play according to what they are told.

The players have Bayesian rationality, which means that if we look at the final action of a certain player, their payoff conditioned on the fact that that was the information that they received from the correlation device must be greater than the payoff they could have achieved if he played something else, given the same information:

$$\sum_{\{\omega|h_i\}} u_i(s_i(\omega), s_{-i}(\omega)) p(\omega|h_i) \geq \sum_{\{\omega|h_i\}} u_i(\bar{s}_i(\omega), s_{-i}(\omega)) p(\omega|h_i), \tag{8}$$

which must be valid for all players i, all information structures $h_i(\omega)$ and all pure strategies $\bar{s}_i(\omega)$. We say that the players are Bayes rational towards the state of the world ω.

If we sum over $h_i(\omega)$ after having multiplied with the corresponding probability, we arrive at

$$\sum_{\omega} u_i(s_i(\omega), s_{-i}(\omega)) p(\omega) \geq \sum_{\omega} u_i(\bar{s}_i(\omega), s_{-i}(\omega)) p(\omega). \tag{9}$$

When these conditions are met, $s(\omega)$ is the final distribution in equilibrium, which means that it is the same distribution as the correlation probabilities, that the players always follow. Hence this is the correlated equilibrium distribution.

The condition in Eq. (8) states that the players only need to maximize their payoff for the information they possess in a particular moment, without considering alternative information, the latter happening in eq. (9). The conditions that arise from eq. (8) are the correlated equilibrium conditions. In the above example, we have for player 1 only that $\bar{s}_1 = D$, so the condition is

$$p(CC)\left[u_1(C,C) - u_1(D,C)\right] + p(CD)\left[u_1(C,D) - u_1(D,D)\right] \geq 0.$$

CORRELATED GAMES IN GAME THEORY

A. Response Probabilities

We introduce a new game that the players engage in, this time about playing for or against the advised move, that we call "correlated game". We retain the notion of the correlation device and without loss of generality assume we only have two players, such that the initial correlations are given by $p(\omega')$, with $\omega' \in \{\mu'\nu'\}$ and thus μ' the available instructions for player i, ν' the available instructions for player $-i$, and both $\mu', \nu' \in \{C, D\}$. In this new game, the "pure" strategies available to the players are to either follow the indications or to not follow them, i.e., $F_{\mu'}$ and $NF_{\mu'}$. We assign independent probabilities to the players following or not following the correlation device, $P_{F_{\mu'}} = 1 - P_{NF_{\mu'}}$, which we call the *response probabilities.* Each instruction that the players can obey creates a new probability variable. A response strategy is defined as $\rho(\omega') = \{P_{F_{\mu'}}, P_{F_{\nu'}}\}$. The final distribution of outcomes is represented by a renormalized correlated strategy with $\omega \in \{\mu\nu\}$ and

$$p^R(\omega) = \sum_{\omega'} P_{\mu,\mu'} P_{\nu,\nu'} p(\omega'), \tag{10}$$

where the transition probability

$$P_{\mu,\mu'} = \delta_{\mu,\mu'} P_{F_{\mu'}} + (1 - \delta_{\mu,\mu'})\, P_{NF_{\mu'}} \tag{11}$$

is a function of $P_{F_{\mu'}}$.

With this formulation, we allow for a continuum of reactions from the players to the correlations. The average payoff of player i is given by

$$\langle u_i \rangle^R = \sum_{\omega} u_i(\omega) p^R(\omega) \equiv \sum_{\omega'} u_i^R(\omega')\, p(\omega'), \tag{12}$$

with

$$u_i^R(\omega') = \sum_{\omega} u_i(\omega) P_{\mu,\mu'} P_{\nu,\nu'} \tag{13}$$

and $u_i(\omega)$ as shorthand for $u_i(\mathrm{s}_i(\omega), \mathrm{s}_{-i}(\omega))$. This result is also equivalent to calculating the convex combination of the games obtained using $P_{F_{\mu'}}$ or $P_{NF_{\mu'}}$ as the probabilities of playing the recommended pure strategy and averaging the payoffs with $p(\omega')$.

Slope Analysis in Game Theory

We can rewrite the slope conditions derived in our paper in formal game-theoretical notation as

$$\sum_{\omega,\nu'} u_i(\omega) P^*_{\mu,\mu'} P^*_{\nu,\nu'} p(\omega') \geq \sum_{\omega,\nu'} u_i(\omega) P_{\mu,\mu'} P^*_{\nu,\nu'} p(\omega'), \tag{14}$$

where $P^*_{\mu,\mu'} = P_{\mu,\mu'}\left(P^*_{F_{\mu'}}\right)$. Substituting eq. (11), we get

$$\sum_{\omega,\nu'} u_i(\omega)\left[\delta_{\mu,\mu'} P^*_{F_{\mu'}} + (1 - \delta_{\mu',\mu}) P^*_{NF_{\mu'}}\right] P^*_{\nu,\nu'} p(\omega') \geq \sum_{\omega,\nu'} u_i(\omega)\left[\delta_{\mu,\mu'} P_{F_{\mu'}} + (1 - \delta_{\mu,\mu'}) P_{NF_{\mu'}}\right] P^*_{\nu,\nu'} p(\omega'),$$

and after performing the sum over μ we obtain

$$\sum_{\nu,\nu'} P^*_{\nu,\nu'} p(\omega') \left[\left(P^*_{F_{\mu'}} - P_{F_{\mu'}} \right) u_i(\mu'\nu) + \left(P^*_{NF_{\mu'}} - P_{NF_{\mu'}} \right) u_i(\mu\nu) \right] \geq 0$$

$$\Leftrightarrow \left(P^*_{F_{\mu'}} - P_{F_{\mu'}} \right) \sum_{\nu,\nu'} P^*_{\nu,\nu'} p(\omega') \left(u_i(\mu'\nu) - u_i(\mu\nu) \right) \geq 0. \tag{15}$$

The coefficient of $\left(P^*_{F_{\mu'}} - P_{F_{\mu'}} \right)$ is the general form of the slopes that we analyze. Clearly when $u(\mu\nu) > u(\mu'\nu)$ the overall condition is satisfied if player i follows μ' with zero probability, which results in a new equilibrium.

Let us assume $\mu' = C$, meaning that the information that player 1 received was C. Substituting in the above, we obtain

$$\left(P^*_{F_{C_1}} - P_{F_{C_1}} \right) \Big(p(CC) \left[P^*_{F_{C_2}} \left(u_1(CC) - u_1(DC) \right) + P^*_{NF_{C_2}} \left(u_1(CD) - u_1(DD) \right) \right]$$
$$+ \, p(CD) \left[P^*_{F_{D_2}} \left(u_1(CD) - u_1(DD) \right) + P^*_{NF_{D_2}} \left(u_1(CC) - u_1(DC) \right) \right] \Big) \geq 0.$$

If the probabilities of following of both players are equal to 1 we recover the correlated equilibrium condition given as an example in the main part of the paper. This shows that the expected correlated equilibrium is only one of the possible equilibria emerging from the response probabilities.

Response Strategy and Nash Equilibrium

The fact that we do not sum over μ' in eq. 14 highlights that we have an independent condition for every response probability. Nonetheless, since these probabilities are indeed independent, we can sum over μ', resulting in

$$\sum_{\omega,\omega'} u_i \left(s_i(\omega), s_{-i}(\omega) \right) P^*_{\mu,\mu'} P^*_{\nu,\nu'} p(\omega') \geq \sum_{\omega,\omega'} u_i \left(s_i(\omega), s_{-i}(\omega) \right) P_{\mu,\mu'} P^*_{\nu,\nu'} p(\omega')$$
$$\Leftrightarrow \sum_{\omega'} u_i^R \left(\rho_i^*(\omega'), \rho^*_{-i}(\omega') \right) p(\omega') \geq \sum_{\omega'} u_i^R \left(\rho_i(\omega'), \rho^*_{-i}(\omega') \right) p(\omega').$$

Both probabilities in $\rho_i(\omega')$, namely $P_{F_{C_i}}$ and $P_{F_{D_i}}$, are independent and each has the same properties as the probability distributions over the pure strategies that correspond to the mixed strategy distributions. They are convex and compact in a finite-dimensional Euclidean space. By the same token as for the mixed strategy, we can be sure to find a fixed point for the response strategies. More specifically, if P is the space of strategy profiles for P_{F_μ}, which thus has a dimensionality equal to the number of possible values of μ, we can define a function called the "reaction correspondence" r_i that maps each response strategy profile ρ to the set of response probabilities that maximize player's i payoff when his opponents play ρ_{-i}. The reaction correspondence is defined as $r : P \to P$. A fixed point of r exists when the players do not have any incentive to change strategy, meaning that they cannot maximize their payoff function any further. The fixed point ρ^* is such that for each player $\rho_i^* \in r_i(\rho^*)$. Thus, a fixed point of r is a response equilibrium, of the same kind as the Nash equilibrium, but now with more probability distributions associated to each player. The proof follows, therefore, analogously from Kakutani's fixed point theorem.

Response Strategy and Correlated Equilibrium

To show that the final game is in correlated equilibrium, we need to make sure that the players actually obey the renormalized probabilities. If the information partitions of player i in two different states are the same, then what player i has played in that state is the same as what he played in the other, which means that having $h_i(\omega^a) = h_i(\omega^b)$ for two otherwise different states ω^a and ω^b, is equivalent to having $s_i(\omega^a) = s_i(\omega^b)$. The transition probabilities then describe a mapping from following the initial correlation device to following the renormalized correlation device:

$$P_{h_i,h_i'} = \delta_{h_i,h_i'} P_{F_{h_i'}} + (1 - \delta_{h_i,h_i'}) P_{NF_{h_i'}}. \tag{16}$$

where we abbreviate $h_i(\omega')$ to h_i' and $h_i(\omega)$ to h_i. We can rewrite eq. (14) as

$$\sum_{\omega,\{\omega'|h_i'\}} u_i \left(s_i(\omega), s_{-i}(\omega) \right) (P^*_{h_i,h_i'} - P_{h_i,h_i'}) P^*_{h_{-i},h'_{-i}} p(\omega'|h_i') \geq 0. \tag{17}$$

Introducing eq. (16) in eq. (17) we get

$$\sum_{\omega,\{\omega'|h_i'\}} u_i\left(s_i\left(\omega\right), s_{-i}\left(\omega\right)\right)\left(\delta_{h_i,h_i'}\left(P^*_{F_{h_i'}} - P_{F_{h_i'}}\right) + \left(1-\delta_{h_i,h_i'}\right)\left(P^*_{NF_{h_i'}} - P_{NF_{h_i'}}\right)\right) P^*_{h_{-i},h'_{-i}} p(\omega'|h_i') \geq 0.$$

Now we realize that the sum over ω is equivalent to a sum over h_i and h_{-i}. Summing only over h_i, we make the transition probabilities effectively act on the payoff functions as

$$\sum_{h_{-i},\{\omega'|h_i'\}} \Bigg(\underbrace{\left(P^*_{F_{h_i'}} - P_{F_{h_i'}}\right) u_i\left(s_i\left(\omega'\right), s_{-i}\left(\omega\right)\right)}_{h_i=h_i'} + \underbrace{\left(P^*_{NF_{h_i'}} - P_{NF_{h_i'}}\right) u_i\left(s_i\left(\bar{\omega'}\right), s_{-i}\left(\omega\right)\right)}_{h_i\neq h_i'} \Bigg) P^*_{h_{-i},h'_{-i}} p(\omega'|h_i') \geq 0.$$

Simplifying, we arrive at an equation analogous to eq. (15), namely

$$\left(P^*_{F_{h_i'}} - P_{F_{h_i'}}\right) \sum_{h_{-i},\{\omega'|h_i'\}} \left[u_i\left(s_i\left(\omega'\right), s_{-i}\left(\omega\right)\right) - u_i\left(s_i\left(\bar{\omega'}\right), s_{-i}\left(\omega\right)\right)\right] P^*_{h_{-i},h'_{-i}} p(\omega'|h_i') \geq 0. \tag{18}$$

We now turn our attention to the coefficients of $\left(P^*_{F_{h_i'}} - P_{F_{h_i'}}\right)$. Looking at the extreme values of $P^*_{F_{h_i'}}$, we can make the following observations:

- $P^*_{F_{h_i'}} = 1$: $\left(P^*_{F_{h_i'}} - P_{F_{h_i'}}\right)$ is non-negative, such that its coefficient must be positive. In this case, $P^*_{h_i,h_i'} = \delta_{h_i,h_i'}$, which means that we can multiply by this factor and reinstate the sum over h_i and substitute ω' by ω and $\bar{\omega'}$ by $\bar{\omega}$;

- $P^*_{F_{h_i'}} = 0$: $\left(P^*_{F_{h_i'}} - P_{F_{h_i'}}\right)$ is non-positive, such that its coefficient must be negative. To have the sum be positive again, we swap the signs of the payoff terms. Multiplying by $P^*_{h_i,h_i'} = 1-\delta_{h_i,h_i'}$ and again summing over h_i, we have the same expression as in the previous case, as now we need to substitute $\bar{\omega'}$ by ω and ω' by $\bar{\omega}$;

- $0 < P^*_{F_{h_i'}} < 1$: $\left(P^*_{F_{h_i'}} - P_{F_{h_i'}}\right)$ can be either negative, positive or zero, which means that its coefficient has to be equal to zero. Since that is the case, it is irrelevant which sign the payoff terms have, and so we can multiply by $P^*_{h_i,h_i'}$ and sum over h_i freely. We then arrive again at the same formula, if we make either one of the substitutions of the previous cases, but now equating to zero.

With this, we can thus rewrite the slope as

$$\sum_{\omega,\{\omega'|h_i'\}} P^*_{h_i,h_i'} P^*_{h_{-i},h'_{-i}} p(\omega'|h_i') \left[u_i\left(s_i\left(\omega\right), s_{-i}\left(\omega\right)\right) - u_i\left(s_i\left(\bar{\omega}\right), s_{-i}\left(\omega\right)\right)\right] \geq 0, \tag{19}$$

for any $\bar{\omega}$. Because the sum is not over h_i', the product of probabilities is equivalent to the conditioning of the renormalized probability distribution on h_i', which leads to

$$\sum_{\{\omega|h_i'\}} p^R(\omega|h_i') \left[u_i\left(s_i\left(\omega\right), s_{-i}\left(\omega\right)\right) - u_i\left(s_i\left(\bar{\omega}\right), s_{-i}\left(\omega\right)\right)\right] \geq 0, \tag{20}$$

such that if the response strategies are in Nash equilibrium, then the final distribution is Bayes rational towards the state of the world ω. Multiplying by $P_{h_i'}$ and summing over h_i' gives

$$\sum_{\omega} p^R(\omega) \left[u_i\left(s_i\left(\omega\right), s_{-i}\left(\omega\right)\right) - u_i\left(s_i\left(\bar{\omega}\right), s_{-i}\left(\omega\right)\right)\right] \geq 0. \tag{21}$$

If the target final distribution is the same as that of $s(\omega)$, then the action corresponding to playing accordingly to a different final distribution is equivalent to playing a different action in S_i with respect to the final distribution, such that $s_i\left(\bar{\omega}\right) = \bar{s}_i(\omega)$, and we arrive at

$$\sum_{\omega} p^R(\omega)\left[u_i\left(s_i\left(\omega\right), s_{-i}\left(\omega\right)\right) - u_i\left(\bar{s}_i(\omega), s_{-i}\left(\omega\right)\right)\right] \geq 0, \tag{22}$$

which is a game with a renormalized distribution that is in correlated equilibrium. With this we can conclude that if the response strategies are in equilibrium, then the final distribution is a correlated equilibrium.

The players are Bayes rational towards the initial world when they both want to follow, since $\omega = \omega'$. When that is not the case, the response probabilities allow them to find a world towards which they want to be rational. Thus we see that the response probabilities create a condition for which the main theorem in [2] applies.

[1] J. F. Nash *et al.*, Proceedings of the national academy of sciences **36**, 48 (1950).

[2] R. J. Aumann, Econometrica: Journal of the Econometric Society , 1 (1987).
